# Proactive and Reactive Transmission Power Control for Energy-Efficient On-Body Communications

**DOI:** 10.3390/s150305914

**Published:** 2015-03-11

**Authors:** Mónica Vallejo, Joaquín. Recas, José L. Ayala

**Affiliations:** 1 Facultad de Minas, Departamento de Energía Eléctrica y Automática, Gaunal, Universidad Nacional de Colombia, Medellín, Carrera 80 No.65-223, Núcleo Robledo, Medellín, 4-72, Colombia; 2 Department of Computer Architecture and Automation, Complutense University of Madrid, C/ Profesor José García Santesmases, Madrid s/n. 28040, Spain; E-Mails: jrecas@fdi.ucm.es (J.R.); jayala@fdi.ucm.es (J.L.A.)

**Keywords:** wireless body sensor networks (WBSNs), transmission power control (TPC), received signal strength indication (RSSI), human body communication

## Abstract

In wireless body sensor network (WBSNs), the human body has an important effect on the performance of the communication due to the temporal variations caused and the attenuation and fluctuation of the path loss. This fact suggests that the transmission power must adapt to the current state of the link in a way that it ensures a balance between energy consumption and packet loss. In this paper, we validate our two transmission power level policies (reactive and predictive approaches) using the Castalia simulator. The integration of our experimental measurements in the simulator allows us to easily evaluate complex scenarios, avoiding the difficulties associated with a practical realization. Our results show that both schemes perform satisfactorily, providing overall energy savings of 24% and 22% for a case of study, as compared to the maximum transmission power mode.

## 1. Introduction

Wireless body sensor networks (WBSNs) are a special case of wireless sensor networks (WSNs) that consist of multiple nodes to be attached to clothing, on the body or even implanted under the skin. Their main functionality is to sense, process and transmit a dataset of measured vital signals to a base station for the monitoring and the healthcare of chronic patients or the tracking of professional sportsmen's performance. In general, the sensor nodes used in these networks are tiny devices characterized by limited memory and processing resources, as well as strong battery power constraints. Therefore, an efficient use of resources is mandatory for maximizing the life time of sensors, while guaranteeing the good performance and high reliability of these networks.

The power consumed by sensor nodes in a WBSN depends on the particular medical application and the transmission channel. According to the applications and depending on the measured variables, data transmission in WBSNs can require different sampling and data transmission rates. Therefore, for slow biosignals, sensor nodes transmit few data, and it is possible to reduce the energy consumption of the transceiver by turning it to the sleeping mode. However, if the biosignals are continuously time varying, sensor nodes transmit a larger volume of data, and hence, the power consumption of the transceiver will be increased.

On the other hand, in a WBSN scenario, the human body plays an important role in the performance of the communication, where the on-body link can be highly dynamic due to the following reasons: (i) the human body introduces temporal variations in the quality of the wireless link, because body movements and postures can change the direction of the antennas, causing detuning and distorted radiation pattern [1]; (ii) it can also obstruct the propagation of the signal, causing non-line-of-sight (NLOS) conditions in an intermittent way [2]; (iii) it introduces attenuation due to the signal absorption by the tissue, dissipating heat [3,4]; (iv) it causes fluctuation in the path loss that can reach 30 dB on average [5].

Due the variability of on-body links and the application characteristics, the use of fixed transmission power can be inadequate. Then, data transmission at high power levels guarantees reliable links, but can result in an unnecessary energy waste. On the other hand, transmitting at low power levels provides energy savings, but at the expense of reducing the reliability and increasing retransmissions. Therefore, there is a trade-off between the energy consumption and the link reliability, and the transmission power should be adaptive in an energy-efficient fashion according to the current state of the link.

The transmission power control techniques allow one to tune the power levels dynamically according to the changing conditions of the links. Therefore, these techniques allow meeting the reliability constraints, while at the same time saving energy. In this paper, we extend our previously proposed transmission power control algorithms ([6,7]) to a complex and realistic scenario. For that purpose, a state-of-the-art WBSN simulator has been upgraded with our model of the biologic channel and an efficient implementation of our reactive and predictive control techniques. The obtained results validate our optimization mechanism and show how our proposals can be used efficiently in deployed WBSNs, where the movement and position of the human subjects affect the transmission properties.

Our main contributions are:

- An extensive experimental data base of link quality metrics, which includes a human sample with diverse body features and different postures.
- An extensive statistical analysis of the impact on the link quality metrics and their relation with the body shape and the body composition, from our experimental database.
- An extensive validation by simulation of the transmission power control policies proposed, thanks to an enhanced version of the Castalia simulator.

The remainder of the paper is organized as follows: In Section 2, we review the related work on transmission power control algorithms for WBSNs. In Section 3, we describe the experimental setup used for the characterization of on-body channel quality in human subjects, showing how the extensive dataset collected in this phase is used in the implementation of our algorithms. In Section 4, we present the ANFIS (Adaptive Neuro-Fuzzy Inference System) link quality estimator model, which is the fundamental unit of our predictive approach, evaluated in the final section. In Section 5, we present an overview of our reactive and predictive policies for transmission power control in WBSNs. Section 6 covers a brief description of the Castalia software used in the validation and simulation of both transmission power control algorithms. In Section 7, we analyze the simulation results. The final section summarizes the conclusions and discusses future works.

## 2. Related Work

A key issue in WBNs is to reduce the energy consumption of the sensor nodes for extending battery lifetime. On average, processing data consumes less power than transmitting the data wirelessly [8], whose power consumption is affected by the speed and the amount of data transmitted. In this context, the use of power optimization methods at multiple layers of the communication stack is nowadays one of the most important research issues in this field; and specifically, the strategies of transmission power control (TPC) are being widely researched, because they ensure an optimal trade-off between energy consumption and reliability requirements.

TPC techniques allow one to select the minimum transmission power level required to achieve good performance within a communication system. The use of these techniques, besides reducing the transmission power consumption, also allows one to reduce the interference problems and to reduce the average contention at the MAC layer.

Different TPC schemes have been proposed for different communication networks, including WBSNs. The type of TPC scheme most frequently used is the link quality-based scheme. Typically, this scheme consists of a closed execution loop between the transmitter and receiver nodes. The loop starts when the transmitter node sends a data packet, and after that, the receiving node takes the measures of the received signal strength indicator (RSSI) value as a quality metric of the communication link. At this point, if the measured RSSI is out of the previously-defined target RSSI margin, the receiving node computes a new transmission power level using a particular TPC algorithm. Finally, the receiver node sends a control packet specifying this new power value to the transmitter node. Some of the most relevant literature in the field is analyzed afterwards.

Xiao *et al.* [9] propose two practical on-line schemes that adapt the transmit power according to the RSSI value obtained from the receiver node (piggybacked in the acknowledgment packet). Both algorithms try to maintain the RSSI of the receiver between the predetermined bounds. In the conservative scheme, if the RSSI drops below the lower configured threshold, then the transmit power is raised to the maximum, and if RSSI is consistently above the configured upper threshold along the last N sample periods, then the transmission level is reduced by a small fixed constant. In the aggressive scheme, the transmitter maintains a running average of the recent RSSI values using the exponential averaging computation, which includes a pre-configured weight value. If this running average exceeds an upper threshold, the transmit level is reduced by a small constant, while if the running average is below a lower threshold, the transmit level is doubled. The algorithms were tested using MicaZ with the CC2420 radio and their Toumaz Sensium Digital Plaster platform.

The results show that in a fast walking scenario, the conservative scheme preserves reliability and yet reduces energy consumption by 1.3% on average when compared to using maximum transmit power. The aggressive scheme saves 23.4% energy on average, at the expense of slightly increased loss. In the resting scenario, the energy savings under both schemes are substantial compared to using maximum power (18.6% and 25.4%) However, some drawbacks of these schemes are the power consumption cost associated with the listening of the feedback packets and, on the other hand, the change in characteristics that the wireless channel can suffer between transmission and feedback and between the feedback and next transmission.

In [10], the authors propose a class of adaptive power control protocols, where the period between each feedback transmission is adaptively varied between 2 and 64 s to accommodate run-time variation in the quality of each channel. The algorithm was tested using a CrossBow MicaZ platform with the CC2420 radio under two different scenarios, a subject walking and resting. The results show that the sensor node radio draws 15% and 21% less power compared to full-power-no-feedback, respectively, for both environments.

Smith *et al.* [11] propose mechanisms for transmission power control based on channel prediction up to 2 s in the the future. The power control methods are based on large datasets taken from ten human subjects performing every-day activities. Channel sounders with Chipcon CC2500 radios were used for collecting the dataset. The authors affirm that the dynamic transmit power control using this predictor can save between 8%–22% of the energy compared with a constant transmit power of −10 dBm.

Quwaider *et al.* [12] proposed a dynamic power control mechanism, named dynamic postural position inference (DPPI), that performs adaptive body posture inference for optimal power assignments. They assume that the average RSSI values can be modeled approximately as a linear function of the transmission power. The performance of this mechanism was evaluated with the Mica2Mote using radio chip CC100. With one of the proposed PC schemes, DPPI, they can save 43%–50% of the energy for different testing persons, compared to using the maximum transmit power [13]. However, in [14], the authors ensure that the DPPI mechanism predicts the transmission power incorrectly for cases where the link state varies frequently.

Our reactive and predictive approaches for the transmission power control are similar to the work research of Quwaider [12] from the point of view that both rely on the detection of the body position for making a decision about the optimal transmission power level; however, the proposed mechanism for posture detection is very different. For DPPI, the postural position is inferred based on the RSSI measurements at the receiver during runtime, which can fail for cases where the link state varies frequently. In our approaches, the postural position detection is done throughout the deployed accelerometers, which are more accurate. Moreover, every expected position has been completely characterized in terms of signal reception. The idea behind our proposal is to take advantage of the hardware being used already in applications, such as fall detection, abnormal movement detection and posture and human activity detection, but also to provide optimization policies that optimally adjust the power transmission level.

On the other hand, from a detailed review of the literature, we can claim that, to the best of our knowledge, our predictive approach is the first in including the real influence of anthropometric and body composition parameters in the variability of RSSI over on-body channels (both factors place a big impact on the development of optimization policies, as will be shown later). Additionally, the development of our optimization policies has required the acquisition of the largest and most exhaustive collection of experimental data using human subjects in an indoor environment that has been reported so far by researchers.

## 3. Experimental Scenario

We followed a similar experimental methodology to [6], but we extended the sample to a group of 37 people distributed between 13 women and 24 men, aged from 20 to 50 years. This large population sample presents different physical characteristics associated with gender and age. Thus, from the onset of puberty to menopause, women maintain a greater body fat mass percentage than men, despite smaller energy intake per kg lean mass [15], and evidence indicates that estrogens contribute to the gender differences in fat mass and the gestational changes in body composition [16]. Moreover, we should not forget that with age, body fat increases and fat-free mass decreases because of the loss of skeletal muscle; hence, the mean body fat of a 20-year-old man weighing 80 kg is 15% compared to 29% in a 75-year-old man of the same weight [17].

**Figure 1.**
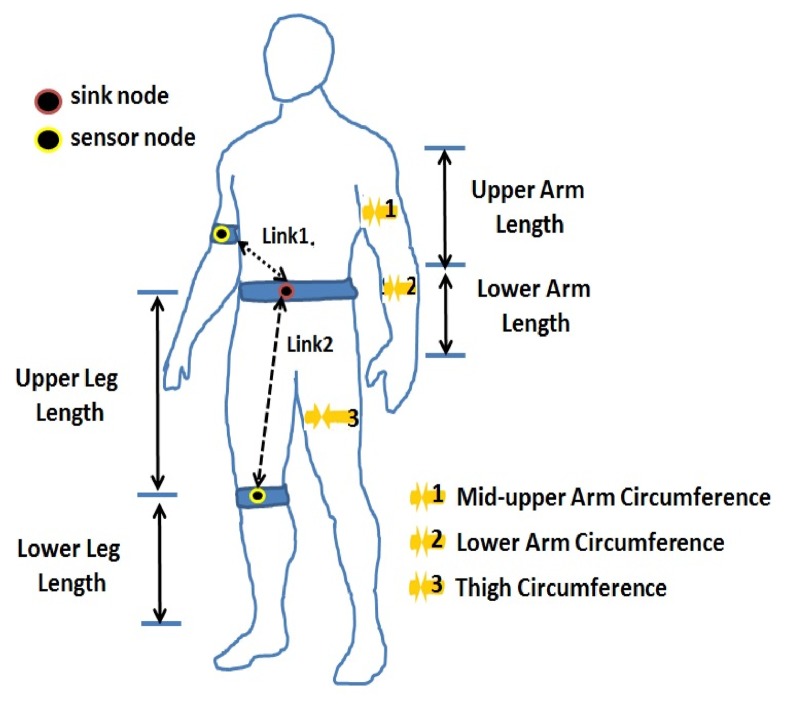
Node location and anthropometric measurements.

For every person, we took anthropometric measurements and body composition values. These parameters, shown in Figure 1, were selected because they are directly related to the locations of the nodes and the links of interest. The measurements of body composition were acquired using the Tanita tetrapolar foot-to-foot bioelectrical impedance analyzer, Model BC-601 [18]. These body composition parameters include: fat mass, muscle mass, bone mass, body mass index, total body water and the levels of body fat percentage and muscle mass of each segment (right/left arm and right/left leg). Body composition and anthropometric parameters allow one to describe the human sample accurately and their expected impact on the radio transmission.

In our experiments, we use the sensor nodes, Shimmer [19]. The Shimmer node is equipped with an ultra-low-power 16-bit microcontroller (TI MSP430), 10 KB of RAM and 48 KB of Flash. This platform includes an IEEE 802.15.4-compliant CC2420 transceiver, which has a sensitivity threshold of −94 dBm and eight programmable power transmission levels from 0 dBm to −25 dBm with a current consumption of 17.4 mA to 8.5 mA, respectively [20]. FreeRTOS has been ported as the real-time operating system in this platform.

The nodes were placed on the subjects' bodies, describing a star topology, with the coordinator placed at the waist (just over the navel) and the node sensors at the right arm (Link L1) and at the right knee (Link L2), as shown in Figure 1. We took measurements of RSSI and the packet error rate (PER); these measurements were gathered for every subject, and these were repeated at every transmission power level available in the radio. All of the results were taken in controlled indoor conditions, under the ground level, to minimize the effect of electromagnetic interference (EMI) (WiFi, 3G, solar radiation, *etc*).

We planned two experimental scenarios to investigate the temporal variations in the quality of two different links in stationary positions:

- Scenario 1: the subject sat in a chair and performed five movements of the arms (Link 1): (1) hands on thighs, denoted as L1/P1; (2) arms crossed, L1/P2; (3) arms extended forward, L1/P3; (4) arms extended up, L1/P4; and (5) arms extended to both sides, L1/P5.
- Scenario 2: the subject sat in a chair and performed four movements of the legs (Link 2): (1) leg at a 90 degree angle with the body, L2/P1; (2) right leg crossed over the left knee, L2/P2; (3) left leg crossed over right knee, L2/P3; and (4) leg extended forward, L2/P4.

As a result, the largest and most complete database (to the author's knowledge) of human channel characterization is built after this experimental work. The univariate analysis of the data has been used in the first descriptive stage of research to know in depth the nature and the meaning, separately, for each one of the variables. Subsequently the study was continued by doing a bivariate analysis to understand how two or more variables relate. Finally, a multivariate analysis was used for explaining the relationships between the variables and RSSI as the link quality metric.

### 3.1. Statistical Analysis

The exploratory statistical analysis was performed using the box plot tool due to its ability to provide relevant statistical information about the input dataset. In Table 1, a concise summary of the quantitative variables is shown.

The dataset of the table has been examined from three main aspects: scatter, symmetry and shape of the data distribution. From the interquartile range (IQR) of the table, a large scatter of the population sample used in the experiments is observed. Specifically, the variables with higher IQR, such as the muscle mass, the body fat mass of the arm, leg and total body fat mass, showed the most dispersive behavior. On the other hand, from Fisher's skewness coefficient, it is possible to affirm that for some variables, such as upper arm length, the lower arm length and the mid-upper arm circumference, the skewness is close to zero, and therefore, they respond to a symmetrical distribution. Some variables, such as bone mass and muscle mass of the arm, of the leg and total muscle mass, show an asymmetrical distribution with a long tail to the left, meaning that these have a negative skew. The other variables show asymmetrical distribution with a long tail to the right, meaning that these have a positive skew. Finally, the kurtosis coefficient quantifies whether the shape of the data distribution matches the Gaussian distribution. A positive kurtosis coefficient, as is shown in Table 1 for all variables, indicates that the observations present a peaked distribution, which is said to be leptokurtic.

The input variable selection is an important part in the construction of any model. In order to build a simpler, reasonable and useful model, correlation tests should be applied among all of the variables of the database, with the purpose of removing the redundancy created by the correlation among variables and, at the same time, reducing the subset of inputs.

Before running this correlation test, it is important to perform a normality test to ensure that the assumptions of a parametric test are met before use. Although, according to [21], for large enough sample sizes (>30 or 40), the violation of the normality assumption should not cause major problems, and therefore, parametric procedures could be used, even when the data are not normally distributed [22]. We prefer checking the assumption, because the validity for parametric tests, such as the correlation test, depends on it. The normality tests used in this study are: the Lilliefors test, Jarque Bera test and Anderson–Darling test contained in MATLAB's Statistics Toolbox. As the results of these test, the following four variables were identified with a non-normal distribution from the overall database: visceral fat level (VFL), body mass index (BMI), muscle mass of leg (MML), muscle mass of arm (MMA).

Due to the variety of distributions shown by the variables, we finally decided to test the overall database, using both Pearson's correlation test and Spearman's correlation test with a statistical significance of *p* < 0.05. The results obtained from both tests were very similar. The results from Pearson's correlation test for the variables related to Link 1 are shown in Table 2. From the table, it is possible to identify three main datasets: with weak correlation (0.5 ≥ *r* > 0), with moderate correlation (0.5 > *r* > 0.8) and with strong correlation (*r* ≥ 0.8).

In conclusion, the following relationships are established from the standpoint of the correlation:

- The variables, lower arm circumference (LAC) and mid-upper arm circumference (MUAC), show a strong correlation between them; we call these Group 1.
- The variables, body fat mass of arm (BFMA), body fat mass (BFM) and total body water (TBW), show a strong correlation between them; we call these Group 2.
- The variables, bone mass (BM), muscle mass (MM) and muscle mass of arm (MMA), show a strong correlation between them; we call these Group 3.

The same statistical analysis was applied for variables related to Link 2. From the results, it is possible to identify two groups of variables strongly correlated amongst each other: Group I, which includes the muscle mass (MM), bone mass (BM) and muscle mass of leg (MML), and Group II, which includes the body fat mass of leg (BFML), body fat mass (BFM) and total body water.

Finally, from the groups of correlated variables that were found, a multivariate analysis was used for explaining the relationships between these and the RSSI as the link quality metric. For this analysis, the highest correlation with the measured RSSI value was used as the selection criteria, and from these results, a set of three and five preliminary variables for each one of the link models was selected.

In summary, from this complete and experimental knowledge and understanding of the behavior of the on-body channel under various scenarios, we are able to describe the effect of several parameters quantitatively on the effectivity of two different transmission power control schemes. These transmission policies are described and profusely evaluated in the next sections. Moreover, the results related to this experimental work can be used in further research on the provision of energy-aware transmission policies.

## 4. Link Quality Estimator Model Based on ANFIS)

Based on our experimental study of temporal variations in the quality of on-body links, described in the previous section, we have proposed a model based on ANFIS (Adaptive Neuro-Fuzzy Inference System) to predict the link quality variations in terms of RSSI. This model has been named the link quality estimator model based on ANFIS (A-LQE). The A-LQE model involves the interaction of input parameters related to the sensor node location (upper or lower body link), the transmission power levels available in the radio, as well as the movement, shape and composition of the human body. This RSSI-prediction model is subsequently used in our proactive policy for the transmission power control.

Below, we shortly describe the A-LQE model; a more profuse description and motivation of the model can be found in [7]. In this paper, we will further exploit the proposed model to develop two reactive and proactive policies for transmission power control, which have been validated in a realistic simulation scenario. Given the complexity of finding an exact analytical formula to predict the RSSI values and in order to build a reasonable model, we have followed a three-phase approach, which is explained next.

- Phase I, feature selection: Once the experimental data have been collected, overall 12 and 13 human body variables are available as input parameters for the models of Link 1 and Link 2, respectively. From the statistical analysis described previously in the Section 3, we selected a set of three and five preliminary variables for each one of the link models.
- Phase II, choice of A-LQE architecture: To assure that the selected input variables are meaningful and descriptive of the output variable (RSSI), we constructed ANFIS models for various combinations of input variables, and then, we chose the one with the best performance (lowest RMSE). The ANFIS models were trained with 1,036 vectors of input data, collected during the experimental work. Seven hundred twenty four vectors (70%) were randomly chosen for the training set, 156 (15%) vectors for testing set and the other 156 (15%) vectors for the validation set. The generalization capability of the models is assured by the proper selection of a large training set. Furthermore, 100 epochs and a training error tolerance of 0.0001 were specified for the training process to assure the achievement of the minimum error tolerance. The performance of the networks is assured for high values of the absolute fraction of variance (*R*^2^), low values of the mean absolute error (MAE) and low values of the root mean squared error (RMSE). Table 3 shows the best ANFIS models obtained for both links.
  For Link 1, the model includes four input parameters: transmission power (Ptx), body position (BPosition), body fat mass (BFM) and mid-upper arm circumference (MUAC). This model has five membership functions of a Gaussian type for every input variable, five rules and one linear output. For Link 2, the model includes three input parameters: transmission power (Ptx), body position (BPosition) and lower leg length (LLL). This model has two membership functions of a triangular type for every input variable, eight rules and one linear output.
- Phase III, validation: For the testing dataset, the validation of the predictive accuracy of the models is analyzed through RMSE, MAE and the average percentage error (APE). From these results, our A-LQE models show satisfactory results with a low APE of 5% and 4.6% for Link 1 and Link 2, respectively.

## 5. Transmission Power Control Algorithms

In this section, we shortly describe our approaches for the transmission power control (TPC approaches) for WBSNs, which were initially introduced in [6,7]. We present again the main ideas of these algorithms, as the work presented here is an experimental validation of both approaches in a realistic simulation scenario, as well as the upgrade of the Castalia simulator to support both TPC policies.

**Figure 2.**
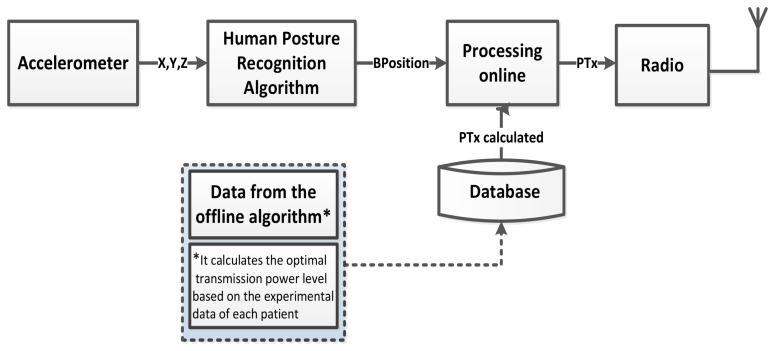
Reactive algorithm for the transmission power control.

Firstly, our reactive algorithm requires that each subject/patient has been previously characterized completely with respect to the RSSI and PER metrics in all scenarios (body positions) and for all transmitted power levels of the radio. Once the communication link is correctly characterized, the computation of the optimal transmission power level is done off-line, using the experimental traces and resulting in a LUT of power levels. The control of the transmission power is done on-line by using the movement detection based on acceleration with low complexity and low overhead (see Figure 2). A detailed description of this algorithm is presented in [6].

**Figure 3.**
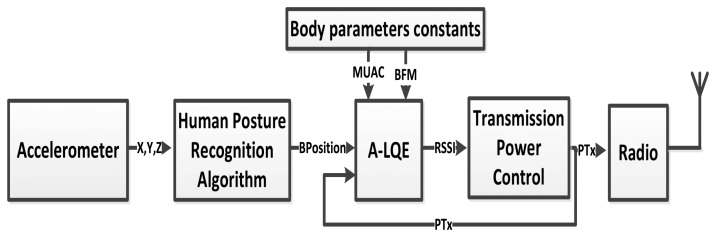
RSSI prediction-based transmission power control approach for Link 1.

Our RSSI prediction-based transmission power control, introduced in [7], consists of two blocks (see Figure 3): an A-LQE model for the specific link, which was explained in Section 4, that allows the prediction of the RSSI variations, and a block named the TPC block, which adjusts the transmission power to the minimum value found experimentally to assure that the RSSI value does not drop below a threshold.

From the RSSI value predicted by the A-LQE model, the TCP block gives as a result an adjusted power level. This power value corresponds to a fixed value that is assigned according to the type of policy chosen (conservative or aggressive) into a range determined from the simulation dataset. In the TCP algorithm, the range of RSSI values are divided into three zones: (1) Zone 1, RSSI values lower than the minimum simulation threshold (Tmin = −80 dBm); (2) Zone 2, RSSI values between −80 dBm and −75 dBm; (3) Zone 3, RSSI values over −75 dBm. The number of zones can be defined by the transmission power levels available in the radio; thus, for a larger number of power levels, a larger number of zones are defined, allowing a finer granularity in the output power.

According to the experimental and simulation results previously obtained, we have decided to implement an aggressive focus for both links, with transmission power levels from 0 dBm to −10 dBm for Link 1 and from 0 dBm to −15 dBm for Link 2. The lowest transmission power level (−25 dBm) was not selected, because there are some critical positions from the point of view of packet loss that do not admit these power values.

## 6. Castalia Simulator

The validation of simulators against real testbeds is not very frequent in the scientific literature, due to the big effort and cost required to implement an extensive set of experimental campaigns. The few authors who have worked on this issue agree on claiming that current simulators are unable to model many essential characteristics of the real world, because these are based on simplified assumptions, and consequently, these simulators cannot produce reliable enough results for real-time scenarios [23–25]. Some authors agree on affirming that these discrepancies are due to the fact that the operating system and layer code execution delays are not taken into account in the simulation models [26].

In this context, the selection of a suitable simulator is a difficult choice. We have selected Castalia as simulation platform for our work, because, among other reasons, it is an open source simulator for WSNs, body area networks (BAN) and common networks composed of low-power embedded devices. It was developed by NICTA (National ICT Australia) as a framework of the OMNeT++ [27] simulator, which has gained wide acceptance in the research community. Castalia includes an average path losses model and a temporal variations model for the modeling of body area networks, which is based on real on-body measurements; additionally, it allows one to use multiple transmission power levels [28], which is a fundamental point for the development of our policies. However, the path loss model included in Castalia does not support the effect on transmission by the anthropometric human characteristics, movement and body positions. Our experimental work has shown the importance of including such information on the path loss model to derive efficient TPC policies. Therefore, Castalia will be upgraded conveniently with our experimental results.

OMNeT++ is a discrete event simulation environment written in C++, suited to supporting frameworks for specialized research fields. The main features offered by Castalia are: an advanced wireless channel model based on empirically-measured data; an advanced radio model based on real low-power radios; extended provisions for modeling sensing and the physical process; a clock-drift model; a power consumption model with state transition for the radio and multiple transmission power levels; MAC and routing protocols, including IEEE 802.15.4 [28]. The basic structure of Castalia is composed of nodes, wireless channel and physical process. These are modules that can communicate through messages sent through the wireless channel [29]. Our research work enhances the simulation capabilities provided by Castalia by extending the set of scenarios available for validating our experimental work and to show the opportunities of energy savings brought by our techniques.

### 6.1. Wireless Channel Model

In the context of body area networks, the experimental data show that the actual path loss may differ very significantly from the average path loss in time. To account for these variations, the current model of Castalia computes the instantaneous path loss of a link as the sum of the average path loss and temporal signal variation at that moment. The spatial variation of the wireless channel (the average path loss) is defined during the channel initialization in the pathlossMap file, which is based on real on-body measurements. On the other hand, the temporal variation of the wireless channel is defined in another file named TemporalModel.txt. For finding the component of the path loss due to temporal variation, Castalia records the last simulated value and the time passed since that value was computed, and from these two numbers, a probability density function (pdf) is generated [28].

The wireless channel model in Castalia has been enhanced with the average path loss measures derived experimentally in our work. In this case, the pathlossMap files that we have generated from experimental measurements implicitly integrate human body mobility and anthropometric information, since each one has a relation to a specific posture and body type. Castalia does not offer a mobility model for BAN; therefore, such an upgrade will facilitate the validation of our energy control policies, which must take into account the posture in order to accurately describe the radio channel energy behavior.

### 6.2. Energy Consumption Model

The resource manager module of Castalia is responsible for calculating the energy use per operation of the node. Castalia models the energy source as an AA battery of 18,720 Joules. Energy is linearly subtracted based on the overall power drawn and simulation time. Modules that model hardware devices send messages to the resource manager in order to signal their power needs [28].

Power consumption in Castalia has two components: radio consumption and baseline consumption. The default baseline consumption value is 6 mW; this corresponds to the energy consumption of a mote when the radio is off and the microcontroller is active. The power consumption corresponding to the radio modes depends on the specific radio chip. Therefore, for sensor nodes based on the CC2420 transceivers, the radio draws 62 mW per second when it is in the listening or receiving state, 1.4 mW when it is in sleeping state, while the power consumption in the transmission state will depend on the transmission power level used.

Differences in energy occur because the radio is ON for different periods of time in each node. The parameters, beacon order (BO) and superframe order (SO), define the duty cycle between the active and inactive periods. In Castalia, BO equals six and SO equals four, while a duty cycle of 25% is the default value for MAC protocol 802.15.4.

In this work, the energy model included in Castalia has been used for evaluating the impact of our transmission power control policies. We have focused on the results related to the transmission term, deriving the results under the application of our TPC policies. Moreover, the path loss model has been closely integrated with the energy model in order to relate our results to the A-LQE approach.

### 6.3. Simulation Setup

We have evaluated our TPC approaches in the Castalia simulation environment. We use Version 3.0 of Castalia on top of Version 4.1 of OMNeT++. A body sensor network of three nodes in a star topology has been defined as our basic simulation scenario in the configuration file, omnetpp.ini, resembling the experimental scenario conducted in our previous work. The sink node always operates at a maximum transmission power level (0 dBm) for ensuring that the sensor nodes receive the beacon. On the other hand, the sensor nodes transmit at a power level selected by the TPC algorithm. The nodes send constant length packets of 25 bytes at a predefined rate of 20 data packets per second to the sink node. Each simulation lasts 52 s, and results are obtained from the average of 10 simulation results.

In Castalia, the radio collision model is configured according to the InterfModel parameter, which can take three different levels: Level 0, 1 and 2. In Level 0, the simulator assumes no collision at all. In Level 1, it considers that the collision happens if more than one node is sending data at the same time and the signal is over the signal interference ratio (SIR). In Level 2, it considers the strongest signal and adds all other signals as noise; then, based on the SIR, the signal is received or ignored [28]. We have used for this study Level 0 with the purpose of reproducing the the experimental scenario most exactly as possible, where each link was tested independently, with no inter-link interference.

We feed the Castalia simulator with the traces of path loss calculated from the RSSI measures obtained with the Shimmer nodes in our experimental scenarios. We define different path loss map files corresponding to each human posture and each transmission power level.

Due to the large number of simulations required, we made extensive use of scripting to automatically generate the parameter description files and to measure and collect the results from the trace files that list for each sensor node the temporal evolution of the signal detected at the node location.

We present the simulation parameters for the nodes: the CC2420 radio file with the default values provided by the simulator; 802.15.4 MAC with two transmission attempts; the temporal variation model to recreate the dynamics of the path-loss fluctuations; and the no collisions model. Table 4 shows the threshold values and other simulation parameters.

### 6.4. Simulation Validation

**Figure 4.**
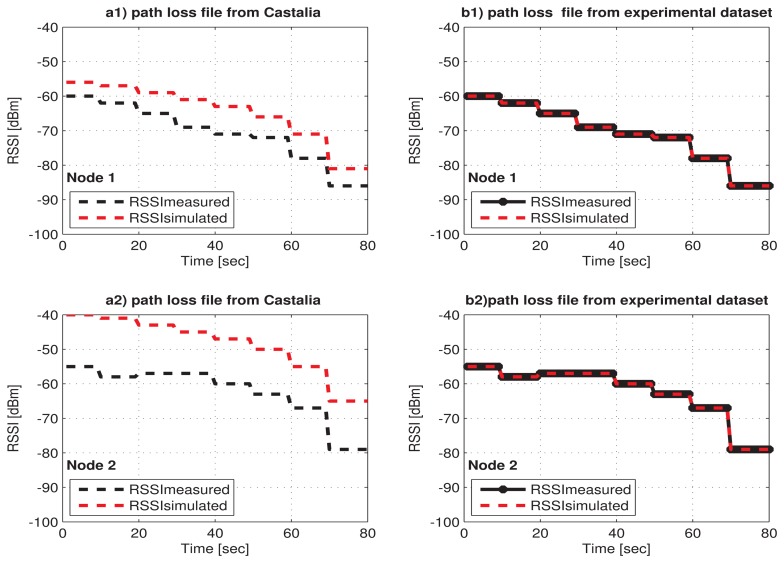
RSSI behavior using the path loss default file of Castalia vs. the path loss file from the experimental dataset.

As previously mentioned, for doing our simulation, the Castalia simulator was extended with the traces of path loss calculated from the RSSI measures obtained with the Shimmer nodes in our experimental scenarios. Different path loss map files corresponding to each human posture and each transmission power level were included in Castalia. The integration of the experimental database in Castalia has allowed ensuring that the RSSI behavior given by the simulator will be more approximate to reality compared with the case when the path loss default file of Castalia is used, such as is shown in the case study of Figure 4 for a1 and a2 in comparison with b1 and b2.

## 7. Simulation Results

In this section, the simulation results obtained with the implementation of our TPC policies in Castalia are presented. In particular, five experimental human subjects have been considered to show how our TCP policies perform. We have tested all possible combinations of movements that simultaneously combine the positions of Scenarios 1 and 2 described previously in Section 3.

We use two metrics for the evaluation of the results obtained with both algorithms: the energy savings in data transmission and the packet error rate (PER). These metrics are recorded at the end of each simulation experiment for each node.

### 7.1. Reactive Algorithm

The reactive algorithm computes the optimal transmission power levels for each posture and each subject using the previously collected experimental dataset. We will call this algorithm “optimal reactive”. On the other hand, we are also interested in testing how the optimal transmission power levels chosen for the human Subject A work when applied to another human subject, B. Therefore, we are interested in analyzing the generalization capabilities of the policy; we will call this algorithm “not optimal reactive”.

The evaluation of both algorithms is done selecting two cases from all of the posture sequences simulated: the best case and a complex case of study that was previously presented in [7]. The best case is referred to as the sequence that showed the greatest energy savings; typically, this sequence includes postures for which Link 2 has a direct line of sight (L2P1, L2P2), and therefore, it allows one to play with a wide range of power values. The case study is the combination of the following postures: L1/P1 + L2/P4, L1/P2 + L2/P3, L1/P3 + L2/P1, L1/P4 + L2/P2 and L1/P5 + L2/P1 (see the description of each one in Section 3).

Table 5 shows the results for the best case under the “optimal” and “not optimal” reactive policy. For the algorithm “not optimal”, data of an extra human subject relative to the five test subjects were chosen randomly. All of the values presented in the table are average values of five subjects during a full sequence of five postures. As can be seen, the optimal algorithm allows one to obtain an energy savings of 29% with an average PER of 5.6% and 1.4% for the sensor nodes of Link 1 and Link 2, respectively. For the “not optimal” case, the average PER is similar to the previous case, 5.5% and 1.4%, respectively, but with a reduction in energy savings of almost 6% with respect to the “optimal” case. These results show the good energy savings achieved by the reactive policy only for previously characterized subjects; therefore, the reactive policy lacks generalization capabilities and requires a characterization and tuning phase per user.

### 7.2. Predictive Algorithm

**Figure 5.**
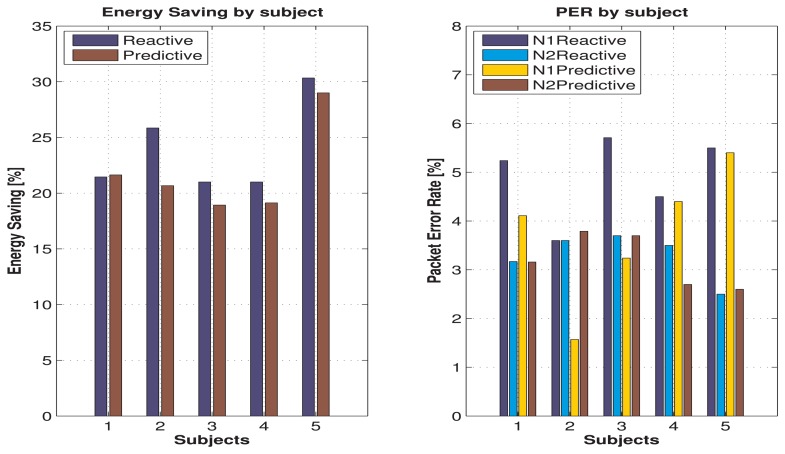
Energy savings and packet error rate for the case study.

Figure 5 shows the results for energy savings and PER in relation to the study case (complex sequence of movements) for each human subject. It can be seen that, from the energy savings point of view, the reactive algorithm shows the best results for all subjects. However, the difference with the results given by the predictive algorithm is relatively small, being 5% for the most significant case (Subject 2) and only 2% for the others. With respect to PER values, we can see that for both algorithms, it is always lower than 6% for all subjects.

Table 6 shows the average simulation results for the five subjects previously mentioned. In summary, the reactive algorithm shows an average energy savings better in only 2% with respect to the predictive algorithm, and on the other hand, the predictive algorithm shows an average PER lower in only 1.4% for Link 1 and 0.1% for Link 2, as compared to the reactive algorithm. Therefore, both algorithms provide similar benefits in terms of energy savings and PER, but with respect to the generalization capabilities, the predictive algorithm can be applied to a broader set of subjects with similar performance.

### 7.3. Specific Case for a Human Subject

**Figure 6.**
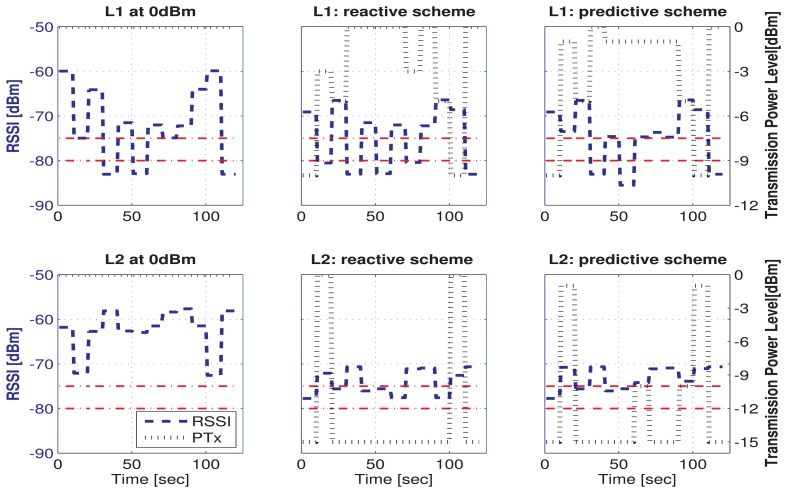
Transmission power and associated RSSI under maximal, reactive and predictive schemes.

Figure 6 shows the selected transmission power and the associated RSSI for a specific human subject doing a sequence of 12 postures under different power control schemes. It can be observed that both algorithms, reactive and predictive, respond appropriately by increasing the power transmission level when the RSSI value drops below the safety threshold (red line in −80 dBm), and they decrease the power transmission level when the RSSI has a value over the upper safety threshold (red line in −75 dBm). The range of RSSI values is divided into three zones delimited by red line thresholds. The minimum RSSI value required to maintain the link reliability is −80 dBm. The aim of the TPC policy is to maintain the RSSI within the safety region defined by the red line thresholds, with the minimal energy consumption. The choice of these thresholds is based on the results obtained by our experimental characterization.

All schemes exhibit considerable fluctuation in their transmission power, since the channel conditions vary quite rapidly. We can see that when we use the maximum transmission power level (0 dBm), the RSSI for both links shows a large variation, whereas when the TPC schemes are used, the RSSI can maintain a relatively stable level (which it is translated into an improvement of the QoS for the communication link). Link 1 is the most critical case, because even for the maximum transmission level, the RSSI value can fall under −85 dBm for some postures.

The reactive scheme shows 5.5% and 3.22% average packet loss and energy savings of 9% and 30.4% for Link 1 and Link 2, respectively, in comparison with the maximum transmission power level. The predictive scheme shows 5.1% and 2.5% average packet loss and energy savings of 9.5% and 30.3% for Link 1 and Link 2, respectively, in comparison with the maximum transmission power level. In conclusion, both power control schemes have a similar behavior, and both provide significant energy savings by adjusting the transmission power level to the channel requirements.

### 7.4. Results Discussion

The reactive algorithm has proven to be an efficient mechanism to adapt the transmission to the channel changing requirements and, at the same time, save precious energy. The reactive algorithm computes the optimal transmission power level based on the PER values measured from an exhaustive characterization of each patient. This characterization is performed for every link, all postures and every power level of the radio. Therefore, any new user of the system must face up to this characterization phase, which can be a very tedious process and a time-consuming task for both the patient and the technical staff. The situation can be even more complicated for cases with patients with chronic conditions or patients with reduced mobility.

On the other hand, the reactive algorithm has proven a similar performance and energy savings for adapting the transmission to the changing channel, but this time with the enormous benefit of avoiding the characterization phase per user. Although the building of the A-LQE model of the predictive algorithm was a very time-consuming task for us, as it required a great effort to collect all of the database used for its training, this task was carried out only once and allowed us to set up a model of path loss for a mobile wireless BAN channel. The advantage of the predictive algorithm is that it can be used in any patient without making a new or additional characterization, as our work has shown. This extensive work was finalized by upgrading the simulation capabilities of Castalia with a new path loss model that enables the proactive set up of the power transmission level.

Both algorithms show acceptable PER values, lower than 6%, and although the energy savings obtained by the predictive strategy are slightly lower than the reactive ones, they represent more than a 22% energy savings. These results could still be improved by tuning the safety RSSI thresholds defined for the TPC block. The obtained results are of great interest in terms of battery autonomy and user satisfaction and can be implemented with low cost and penalty, as they exploit the multiple transmission power levels provided by current radio chips.

## 8. Future Works

The exploration of the space of solutions is considered as future work by running the simulations with varying values for parameters, such as duty cycle, data rate, packet size and the collision model. This analysis would provide the evaluation of the real impact of these parameters on the performance of the TPC policies. Furthermore, we are interested in evaluating other approaches based on expert systems for the implementation of the TPC block of the predictive scheme, with the aim of improving the adaptation of the transmission power to the channel requirements.

## Figures and Tables

**Table 1.** Descriptive statistics of anthropometric and body composition variables.

**Variable**	**Minimum**	**Q1**	**Q2**	**Q3**	**Maximum**	**IQR**	**Skewness**	**Kurtosis**
Upper Arm Length (UAL) (cm)	28	31	33	35	38	4	0.09	2.18
Lower Arm Length (LAL) (cm)	22	25	26	27	30	2	0.06	2.67
Upper Leg Length (ULL) (cm)	44	48	53	54	63	6	0.27	2.74
Lower Leg Length (LLL) (cm)	37	40.5	43	45	51	4.5	0.33	2.27
Mid-upper Arm Circumference (MUAC) (cm)	23	28	30	32	37	4	0.04	2.62
Lower Arm Circumference (LAC) (cm)	22	25	27	28	33	3	0.42	2.98
Thigh Circumference (TC) (cm)	35	39	41	44	50	5	0.25	2.97
Muscle Mass (MM) (kg)	34	44.5	57.2	61.6	77.8	17	−0.18	2.10
Bone Mass (BM) (kg)	1.9	2.4	3	3.22	4	0.8	−0.20	2.02
Body Fat Mass (BFM) (%)	5.8	16.6	23.9	29.7	42.2	13	0.095	2.67
Body Fat Mass of Arm (BFMA) (%)	5.1	14.9	20.4	30.2	45.4	15.3	0.302	2.33
Body Fat Mass of Leg (BFML) (%)	6.2	13.4	19.6	30.6	45.3	17.1	0.434	2.12
Muscle Mass of Arm (MMA) (kg)	1.4	2.1	3.2	3.7	4.5	1.6	−0.38	1.89
Muscle Mass of Leg (MML) (kg)	6	7.6	10.2	11.1	13.6	3.5	−0.27	1.97
Body Mass Index (BMI) (kg/m^2^)	19.7	22.2	23.8	28.5	33.1	6.3	0.514	2.12
Total Body Water (TBW) (%)	43.5	51.7	55.4	59.5	67.6	7.7	0.115	2.59
Visceral Fat Level (VFL)	1	1.75	4	7	13	5.2	0.807	2.65

**Table 2.** Correlations between anthropometric and body composition variables: 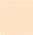 weak correlation; 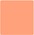 moderate correlation; 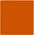 strong correlation.

	**UAL**	**MUAC**	**LAL**	**LAC**	**BFM**	**BFMA**	**MM**	**BM**	**TBW**	**BMI**	**VFL**
**MUAC**	0.25										
	0.13										
**LAL**	0.77	0.23									
	0.00	0.18									
**LAC**	0.56	0.80	0.49								
	0.00	0.00	0.00								
**BFM**	−0.34	−0.00	−0.22	−0.13							
	0.04	1.00	0.20	0.45							
**BFMA**	−0.37	−0.16	−0.22	−0.24	0.96						
	0.02	0.34	0.20	0.16	0.00						
**MM**	0.64	0.63	0.43	0.77	−0.45	−0.55					
	0.00	0.00	0.01	0.00	0.00	0.00					
**BM**	0.63	0.63	0.42	0.77	−0.45	−0.54	1.00				
	0.00	0.00	0.01	0.00	0.00	0.00	0.00				
**TBW**	0.23	−0.11	0.09	−0.02	0.97	−0.92	0.30	0.29			
	0.18	0.52	0.61	0.92	0.00	0.00	0.07	0.08			
**BMI**	0.12	0.54	0.07	0.50	0.30	0.15	0.38	0.36	−0.35		
	0.49	0.00	0.70	0.00	0.07	0.39	0.02	0.03	0.03		
**VFL**	0.02	0.69	0.06	0.54	0.40	0.26	0.45	0.45	−0.53	0.65	
	0.91	0.00	0.72	0.00	0.01	0.12	0.00	0.01	0.00	0.00	
**MMA**	0.59	0.64	0.40	0.75	−0.54	−0.65	0.98	0.98	0.40	0.31	0.40
	0.00	0.00	0.01	0.00	0.00	0.00	0.00	0.00	0.01	0.06	0.02

**Table 3.** Link quality estimator models based on ANFIS (Adaptive Neuro-Fuzzy Inference System) (A-LQE) for both links with the training dataset.

**Link**	**Inputs Name**	**RMSE**	**R2**	**MAE**
L1	Ptx /BPosition /BFM/MUAC	5.38	0.84	4.21
L2	Ptx/BPosition/LLL	4.66	0.85	3.6

**Table 4.** Simulation parameters.

**Parameter Name**	**Simulation Time**	**Radio Model**	**Channel Frequency**	**MAC Protocol**	**Packet Size**	**Number Nodes**	**Sink Node**	**Application**
**Value**	52 s	CC2420	2.4 GHz	MAC 802.15.4	25 bytes	3	Node 0	Throughput test

**Table 5.** Simulation results for the reactive algorithm in the best case. PER, packet error rate. Transmission Power Levels (TPL) , node 1 (n1) and node 2 (n2).

**Reactive**	**TPLn1**	**TPL n2**	**RSSI n1**	**RSSI n2**	**Energy Saving**	**PER n1**	**PER n2**
Optimal	−5 dBm	−15 dBm	−78 dBm	−70 dBm	29%	5.6%	1.4%
Not Optimal	−2 dBm	−15 dBm	−74 dBm	−70 dBm	23%	5.5%	1.4%

**Table 6.** Simulation results for the case study. Transmission Power Levels (TPL) , node 1 (n1) and node 2 (n2).

**Algorithm**	**TPL n1**	**TPL n2**	**RSSI n1**	**RSSI n2**	**Energy Saving**	**PER n1**	**PER n2**
Reactive Optimal	−5 dBm	−10 dBm	−71 dBm	−74 dBm	24%	5.1%	3.3%
Predictive	−3 dBm	−11 dBm	−71 dBm	−73 dBm	22%	3.7%	3.2%
